# Development and Validation of a Machine Learning-Based Dementia Screening Tool: The Six-Question Dementia Screening Test

**DOI:** 10.1177/15333175261424333

**Published:** 2026-02-17

**Authors:** Meng-Tien Wu, Kuan-Ying Li, Ching-Fang Chien, Ling-Chun Huang, Chen-Wen Yen, Yuan-Han Yang

**Affiliations:** 1 School of Post-Baccalaureate Medicine, College of Medicine, 164790 Kaohsiung Medical University, Kaohsiung, Taiwan; 2 Department of Neurology, Kaohsiung Medical University Hospital, 164790 Kaohsiung Medical University, Kaohsiung, Taiwan; 3 Neuroscience Research Center, 164790 Kaohsiung Medical University, Kaohsiung, Taiwan; 4 Department of Neurology, Kaohsiung Medical University Gangshan Hospital, 164790 Kaohsiung Medical University, Kaohsiung, Taiwan; 5 Department of Mechanical and Electro-Mechanical Engineering, 34874 National Sun Yat-Sen University, Kaohsiung, Taiwan; 6 National Center for Geriatrics and Welfare Research, National Health Research Institutes, Taiwan

**Keywords:** dementia, machine learning, cognitive impairment, neuropsychological assessment, early detection

## Abstract

Timely detection of dementia is crucial for reducing its health and societal burden. Standard tools such as the Mini-Mental State Examination (MMSE) and Cognitive Abilities Screening Instrument (CASI), although widely used, are limited by time and resource demands. This study developed and validated a machine learning–based screening tool using the Six-Question Dementia Screening Test (6Q-DS), a brief interview of six items. Data from 533 older adults at a neurology clinic in Taiwan (331 with dementia, 202 without) were analyzed with eXtreme Gradient Boosting. The 6Q-DS achieved an AUC of 0.936, sensitivity 0.879, specificity 0.951, and accuracy 0.907 for dementia vs non-dementia. For identifying very mild dementia vs non-dementia, the AUC was 0.874, with a sensitivity of 0.818, specificity of 0.805, and accuracy of 0.810. Comparable to MMSE and CASI, the 6Q-DS provides a practical, rapid, and user-friendly tool for dementia screening.

## Introduction

Dementia is a chronic and progressive syndrome that impairs memory, executive function, language, and other cognitive abilities, ultimately compromising independent living.^1^ Its diagnosis in clinical practice typically relies on comprehensive assessments, including medical history, neurological and physical examination, neuropsychological testing, laboratory investigations, and neuroimaging.^2^ However, these procedures are time-consuming, costly, and not easily applicable in primary care or community-based screening programs, where most older adults first present, highlighting the need for brief, accurate, and scalable screening tools that can be administered outside specialized memory clinics.

Early detection in community settings remains a major challenge. Several screening tools have been developed for early-stage detection, but most have notable limitations. The Mini-Mental State Examination (MMSE) is widely used and validated,^3,4^ yet its accuracy is affected by factors such as age, education, and socioeconomic status, and it requires 7-10 min to administer.^5,6^ The Cognitive Abilities Screening Instrument (CASI), which integrates components from the MMSE, Modified MMSE, and Hasegawa Dementia Scale, provides a broader assessment across nine cognitive domains and allows MMSE score derivation.^7^ However, CASI administration takes 15-20 min and requires trained personnel, reducing its feasibility in fast-paced clinical settings. Another tool, the Ascertain Dementia 8-item Informant Questionnaire (AD8) provides a rapid, informant-based assessment^8^ and is useful for tracking intra-individual cognitive change over time. Nevertheless, its utility is restricted when a reliable informant is unavailable, and self-reported versions have shown reduced diagnostic accuracy in both primary care and memory clinic contexts.^9-11^

In recent years, a variety of neuropsychological screening instruments have been used internationally for dementia detection. Beyond global cognitive tests such as MMSE, protocols in Western countries increasingly incorporate functional assessments, including instrumental activities of daily living (IADLs) and financial capacity tasks,^12-14^ as these domains are highly sensitive to early neurocognitive decline. Evidence indicates that changes in financial decision-making and everyday money management are closely linked with neurocognitive disorders and can support differential diagnosis and risk identification.^12,14^ Measures of financial capacity also capture culturally influenced behaviors and vulnerabilities, which have been examined in diverse populations and are increasingly recognized as clinically meaningful outcomes.^12,14^

Parallel to these developments, machine learning (ML) has been increasingly applied across different cultural contexts to improve dementia screening and classification. ML methods have been successfully used with neuropsychological test data to distinguish dementia from healthy aging, to predict progression, and to identify the most informative cognitive and functional indicators. Recent study demonstrates that ML models integrating traditional neuropsychological tests with financial capacity measures or IADL performance can enhance diagnostic accuracy and reduce the number of required test items, making them particularly suitable for primary care and community settings.^14^

Given these limitations, there is a critical need for more efficient, scalable, and accurate tools for dementia screening—particularly those suitable for use in community or primary care settings. In this context, ML offers a promising alternative. ML enables computers to learn from data and make predictions without relying on predefined rules or assumptions.^15^ Unlike traditional statistical approaches, ML algorithms can analyze large, complex datasets and detect nonlinear patterns and interactions that may otherwise go unnoticed.^16^ Recent applications of ML in dementia research have shown encouraging results, including the identification of neuroimaging biomarkers,^17^ the classification of dementia subtypes,^18^ and high-accuracy prediction of dementia onset—achieving up to 92% accuracy in large-scale studies.^19^

ML techniques are generally categorized as supervised or unsupervised.^20^ Supervised learning uses labeled data to train predictive models, while unsupervised learning detects patterns in unlabeled data. Common algorithms include decision trees, support vector machines, random forests, and gradient boosting.^21^ This study employed eXtreme Gradient Boosting (XGBoost), a high-performance ensemble learning algorithm based on gradient boosting.^22^ XGBoost builds sequential decision trees, correcting prior errors and incorporating regularization to prevent overfitting, thus improving generalizability and computational efficiency.^23,24^ It also provides feature importance metrics, making it particularly suitable for clinical applications such as dementia screening.

This study aimed to develop a rapid and practical dementia screening tool suitable for community populations by leveraging machine learning techniques and easily accessible data. The objectives included designing the tool through expert consensus, creating a labeled dataset from clinical data, training supervised ML models, and evaluating their diagnostic performance in comparison with existing screening methods.

## Methods

### Study Population and Diagnostic Criteria

This cross-sectional study included 533 participants (331 with dementia and 202 without), recruited from the Neurology Outpatient Department at Kaohsiung Municipal Ta-Tung Hospital between June 2021 and August 2022. All participants were aged 60 years or older and capable of completing a full neuropsychological assessment. Each underwent a comprehensive evaluation, including medical history, neurological and physical examination, neuropsychological assessments, laboratory tests, and brain imaging (computed tomography or magnetic resonance imaging). Individuals with major psychiatric illness or other neurological disorders unrelated to dementia were excluded. All participants were thoroughly informed about the screening tests, and written informed consent was obtained from each individual. The study received ethical approval from the Institutional Review Board of Kaohsiung Municipal Ta-Tung Hospital.

The sample size in the present study was not determined by a priori power calculation because the primary aim was to develop and validate a machine learning–based dementia screening model rather than to test a single statistical hypothesis. Participants were recruited consecutively from our memory clinic database during the study period, and all eligible cases with complete data were included. This approach maximizes the available information for model training and evaluation, which is particularly important in machine learning applications where larger sample sizes improve model stability and generalizability. The final sample comprised 202 non-dementia participants, 108 individuals with very mild dementia, and 223 with mild to moderate dementia.

Dementia was diagnosed based on the 2011 criteria established by the National Institute on Aging and the Alzheimer’s Association (NIA-AA).^1^ In accordance with the core clinical criteria, a diagnosis of dementia required (1) cognitive decline from a previous level of functioning, (2) impairment in at least one cognitive domain documented by history and/or testing, (3) interference with independence in daily activities, and (4) an insidious onset with gradual progression of symptoms. The Clinical Dementia Rating (CDR) was used to stage dementia severity. A CDR score of 0 indicated not demented, whereas scores of 0.5, 1, and 2 corresponded to very mild, mild, and moderate dementia, respectively.^25,26^ Consistent with prior longitudinal clinical research, a CDR score of 0.5 was used to denote very mild dementia.^25^ Participants with a CDR score of 3 (severe dementia) were excluded. Participants in the non-dementia group exhibited no or only mild cognitive decline and did not meet NIA-AA criteria for dementia.

Probable Alzheimer’s disease (AD) was diagnosed according to the NIA–AA core clinical criteria, characterized by insidious onset and gradual progression of cognitive decline that interferes with independence in daily activities, typically with prominent episodic memory impairment, although language, visuospatial, or executive functions may also be affected.^1^ Other dementia subtypes were diagnosed as follows: Parkinson’s disease (PD) with dementia according to 2007 Movement Disorder Society Task Force criteria^27^; dementia with Lewy bodies (DLB) based on 2017 DLB Consortium criteria,^28^ requiring dementia with fluctuating cognition, hallucinations, parkinsonism, or rapid eye movement sleep behavior disorder; and vascular dementia using National Institute for Neurological Disorders and Stroke criteria,^29^ requiring imaging-confirmed cerebrovascular disease with a temporal relationship to cognitive impairment.

### Assessment Tools

#### Six-Question Dementia Screening Test (6Q-DS)

The 6Q-DS is a structured, direct interview composed of six questions derived from the AD8 and CASI, selected by consensus among three senior neurologists with expertise in dementia. The test evaluates domains including mood, memory, temporal orientation, and concentration. The 6Q-DS comprises the following six questions: (1) Are you feeling depressed? (2) Do you say the same things (like questions or stories) repeatedly? (3) Do you think you have a problem with memory or thinking? (4) What is the year? (5) What is the month? (6) Please count down 100 by three for five times (serial 3’s). The first question requires a binary (“Yes” or “No”) response; questions two and three offer three options (“Yes,” “Sometimes,” or “No”); questions four through six are scored as either “Correct” or “Incorrect,” with each step in the serial subtraction task (Question 6) treated as an individual item. In total, 10 items are generated.

#### Cognitive Abilities Screening Instrument (CASI)

The CASI, developed for cross-cultural use, is based on the MMSE, Modified MMSE, and Hasegawa Dementia Screening Scale.^7^ It comprises 25 items covering nine cognitive domains: long-term memory, short-term memory, attention, mental manipulation, orientation, drawing, abstract thinking, category fluency, and language. The total score ranges from 0 to 100, with higher scores indicating better cognitive performance.

#### Estimated Mini-Mental State Examination (MMSE)

The MMSE comprises 11 items that evaluate six cognitive domains: orientation, registration, concentration, short-term memory, language, and visuospatial function.^6^ The maximum total score is 30, with higher scores reflecting better cognitive performance. In this study, an estimated MMSE score was derived using six corresponding domains from the CASI: short-term memory, mental manipulation, orientation, drawing, abstract, and language.^10^

### Statistical Analysis

Demographic data (age, sex, education), CASI scores, and estimated MMSE scores were analyzed. Continuous variables are expressed as mean ± standard deviation, while categorical variables are presented as counts and percentages (n, %). Differences between groups were analyzed using one-way ANOVA and chi-square tests for continuous and categorical variables, respectively. A *P*-value of <0.05 was considered statistically significant. Bonferroni post hoc analysis was conducted for one-way ANOVA tests. Analyses were conducted using IBM SPSS Statistics for Windows, version 20.0 (Armonk, NY, USA).

### Machine Learning Models

A machine learning model for dementia prediction was developed using the XGBoost algorithm, an ensemble method based on decision trees. Ten input features were extracted from responses to the 6Q-DS (Table 1). Participants with missing data were excluded. The dataset was randomly split 100 times into training (80%) and test (20%) subsets to ensure robust evaluation through repeated classification. The model was implemented using Python 3.7.

**Table 1.** Questions in the Six-Question-Based Dementia Screening Test (6Q-DS)

Item number	Question content	Options
D01	Are you feeling depressed?	“Yes” or “No”
M01	Do you repeat the same things (like questions or stories) over and over again?	“Yes”, “sometimes” or “No”
M02	Do you think you have a problem with memory or thinking?	“Yes”, “sometimes” or “No”
TO01	What is the year?	“Correct” or “incorrect”
TO02	What is the month?	“Correct” or “incorrect”
C01	1st calculation for serial 100-3	“Correct” or “incorrect”
C02	2nd calculation for serial 100-3	“Correct” or “incorrect”
C03	3rd calculation for serial 100-3	“Correct” or “incorrect”
C04	4th calculation for serial 100-3	“Correct” or “incorrect”
C05	5th calculation for serial 100-3	“Correct” or “incorrect”

Model performance was primarily evaluated using the area under the receiver operating characteristic curve (AUC), classified as excellent (0.9-1.0), good (0.8-0.9), acceptable (0.7-0.8), and poor (0.6-0.7).^30^ Additional metrics included sensitivity (recall), specificity, accuracy, positive predictive value (PPV), negative predictive value (NPV), F1-score, and Matthews correlation coefficient (MCC).^31,32^ These metrics were computed using standard formulas based on true positives (TP), true negatives (TN), false positives (FP), and false negatives (FN). Sensitivity was defined as TP/(TP + FN), specificity as TN/(TN + FP), and accuracy as (TP + TN)/(TP + TN + FP + FN). Precision (PPV) was defined as TP/(TP + FP), and NPV as TN/(TN + FN). The F1-score was used to balance precision and recall, while MCC, a robust metric for imbalanced datasets, was calculated as:

$$\left(\text{TP}\times \text{TN}\;–\;\text{FP}\times \text{FN}\right)\;/\sqrt{\left(\text{TP}+\text{FP}\right)*\left(\text{TP}+\text{FN}\right)*\left(\text{TN}+\text{FP}\right)*\left(\text{TN}+\text{FN}\right).}$$

## Results

### Demographics

A total of 533 participants (186 men and 347 women), with a mean age of 77.6 ± 8.2 years, were recruited. Among them, 108 (20.3%) were classified as having VMD (CDR = 0.5), and 223 (41.8%) were identified with mild-to-moderate dementia (MiD) (CDR ≥1). Of the 331 participants with dementia (CDR ≥ 0.5), 308 (93.1%) were diagnosed with probable AD, followed by Parkinson’s disease dementia (11, 3.3%), probable DLB (8, 2.4%), and vascular dementia (4, 1.2%). Table 2 presents the baseline demographic and cognitive profiles of the participants. Although the three cognitive groups differed significantly in baseline demographics—particularly age (*P* < 0.001) and education (*P* < 0.001)—with the MiD group being oldest and least educated, these differences reflect the natural progression of dementia and are commonly observed in clinical cohorts. Sex distribution showed marginal significance across groups (*P* = 0.048, chi-square test). Bonferroni post-hoc analyses confirmed specific pairwise differences while controlling for multiple comparisons. The estimated total scores on the estimated MMSE and CASI were significantly lower in the MiD group than in the other two groups (both *P* < 0.001). These demographic variations do not compromise the validity of the reported machine learning analyses, as the XGBoost algorithm inherently accounts for covariate interactions through its tree-based ensemble structure and regularization techniques. Model performance was evaluated using robust 100-repeated 80/20 train-test splits and the MCC, a balanced metric suitable for imbalanced datasets without assuming group homogeneity.

**Table 2.** Comparison of Demographic Data Among Groups With Different Stages of Cognitive Impairment

Groups	Non-dementia (ND)	CDR = 0.5 (VMD)	CDR ≥ 1 (MiD)	*P* value
N	202	108	223	
Age, year	72.1 ± 6.6	78.5 ± 6.5	82.1 ± 7.3^a^	<0.001^***^
Female, n (%)	126 (62.4)	63 (58.3)	158 (70.9)	0.048^*^
Education, year	11.1 ± 4.6^b^	9.2 ± 4.8	6.3 ± 5.1	<0.001^***^
Estimated MMSE	25.8 ± 5.2^b^	22.1 ± 4.5	12.6 ± 6.0	<0.001^***^
CASI	87.5 ± 11.4^b^	76.1 ± 13.5	42.6 ± 20.6	<0.001^***^

CASI, Cognitive Abilities Screening Instrument; CDR, Clinical Dementia Rating; MiD, mild to moderate dementia; ND, non-dementia; VMD, very mild dementia.

*Chi-square analysis, *P* < 0.05.

***One-way ANOVA, *P* < 0.001.

^a^Bonferroni post hoc analysis showed MiD > VMD > ND.

^b^Bonferroni post hoc analysis showed ND > VMD > MiD.

### Diagnostic Performance in Dementia (CDR ≥ 0.5) Versus Non-Dementia

**Figure 1.**
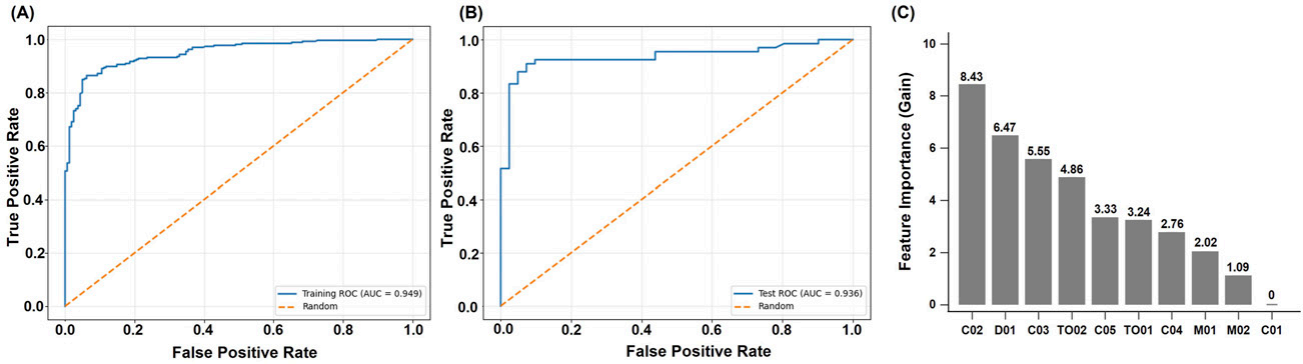
(A) ROC curve of the 6Q-DS for distinguishing dementia from non-dementia in the training set (AUC = 0.949). (B) ROC curve for the same classification in the test set (AUC = 0.936). (C) Feature importance of the 6Q-DS model for detecting dementia

The 6Q-DS demonstrated strong discriminative performance using the XGBoost algorithm. In the training set, the model for distinguishing dementia from non-dementia achieved an accuracy of 0.887, sensitivity of 0.864, specificity of 0.925, and an AUC of 0.949. The F1 score, MCC, PPV, and NPV were 0.905, 0.772, 0.950, and 0.805, respectively. In the test set, performance remained robust, with an accuracy of 0.907, sensitivity of 0.879, specificity of 0.951, and an AUC of 0.936. The F1 score was 0.921, MCC was 0.813, PPV was 0.967, and NPV was 0.830. These results suggest that the 6Q-DS is both accurate and generalizable across datasets. The ROC curves for both the training and test sets further illustrate the model’s strong classification performance, as shown in Figure 1A and B. Overall, the model showed strong performance in identifying dementia cases and moderate reliability in ruling out non-cases. Feature importance (Figure 1C) indicated that the most informative features were the second serial subtraction of 100 minus 3 (C02), the feeling of depression (D01), and the third calculation for serial 100 minus 3 (C03).

### Diagnostic Performance in Very Mild Dementia (CDR = 0.5) Versus Non-Dementia

**Figure 2.**
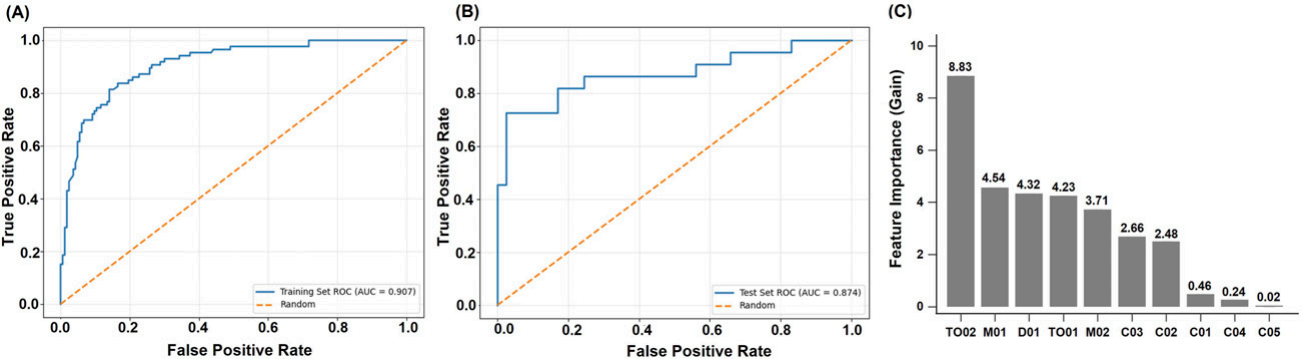
(A) ROC curve of the 6Q-DS for distinguishing very mild dementia from non-dementia in the training set (AUC = 0.907). (B) ROC curve for the same classification in the test set (AUC = 0.874). (C) Feature importance of the 6Q-DS model for detecting very mild dementia

In the training set, the model for distinguishing VMD from non-dementia achieved an accuracy of 0.823, sensitivity of 0.837, specificity of 0.816, and an AUC of 0.907. The F1 score, MCC, PPV, and NPV were 0.766, 0.632, 0.706, and 0.905, respectively. In the test set, performance remained robust, with an accuracy of 0.810, sensitivity of 0.818, specificity of 0.805, and an AUC of 0.874. The F1 score was 0.750, MCC was 0.603, PPV was 0.692, and NPV was 0.892. These results suggest that the 6Q-DS is both accurate and generalizable across datasets. The ROC curves for both the training and test sets further illustrate the model’s strong classification performance, as shown in Figure 2A and B. Overall, the model showed strong performance in identifying VMD cases and moderate reliability in ruling out non-cases. Feature importance (Figure 2C) showed the top three features were temporal orientation for month (TO02), repetitive speech or behavior (M01), and the feeling of depression (D01).

### Comparison of Diagnostic Performance in Dementia (CDR ≥ 0.5) Versus Non-Dementia Across Screening Tools

Table 3 summarized the comparative diagnostic accuracy of the 6Q-DS, estimated MMSE, and CASI in distinguishing dementia (CDR ≥ 0.5) from non-dementia. Using a cutoff value of 23/24, the estimated MMSE achieved the good AUC and the moderate sensitivity for differentiating all levels of dementia from non-dementia (AUC = 0.966, sensitivity = 0.879, specificity = 0.951). Using a cutoff value of 76/77, the CASI achieved the high AUC and the best sensitivity for differentiating all levels of dementia from non-dementia (AUC = 0.989, sensitivity = 0.965, specificity = 0.920). The 6Q-DS exhibited higher sensitivity than the estimated MMSE in differentiating dementia from non-dementia.

**Table 3.** Comparisons of Sensitivity, Specificity, and Area Under the Curve (AUC), as Well as Cutoff Scores for Different Screening Tools

	6Q-DS	Estimated MMSE	CASI
All dementia (CDR ≥ 0.5) vs ND			
AUC	0.936	0.966	0.989
Sensitivity	0.879	0.879	0.965
Specificity	0.951	0.951	0.920
Cutoff	Not applicable	23/24	76/77

6Q-DS, Six-Question Dementia Screening Test; AUC, area under the curve; CASI, Cognitive Abilities Screening Instrument; CDR, Clinical Dementia Rating; MMSE, Mini-Mental State Examination; ND, non-dementia.

## Discussion

In this study, we developed a novel, brief screening tool to distinguish dementia from non-dementia using ML algorithms. The 6Q-DS was designed to capture changes across a broad range of cognitive and non-cognitive domains, including memory, time orientation, concentration, and mood. Utilizing the XGBoost model, this tool demonstrated satisfactory sensitivity (0.879) and specificity (0.951) in differentiating older adults with dementia from those without. It also showed good discriminative performance in identifying VMD, with a sensitivity of 0.818 and specificity of 0.805.

Currently, the MMSE, CASI, and AD8 are widely used dementia screening tools, particularly in Taiwan.^33^ A systemic review that included 102 studies involving 10,263 patients with dementia reported that the MMSE had a pooled sensitivity of 0.81 (95% confidence interval [CI], 0.78-0.84) and a specificity of 0.89 (95% CI, 0.87-0.91) when used to detect dementia.^7^ However, the MMSE is less sensitive in detecting mild cognitive impairment and mild dementia, especially among individuals with higher education levels.^34^ The CASI, a cross-cultural dementia screening tool, has reported sensitivity rates ranging from 0.91 to 0.95 and specificity rates from 0.91 to 0.94.^10^ Based on a cutoff score of ≥2 for the AD8 to detect dementia, the pooled sensitivity was 0.91 (95% CI, 0.89-0.92) and the specificity was 0.78 (95% CI, 0.76-0.80).^35^ Our findings are consistent with prior studies that reported high sensitivity for the CASI in distinguishing dementia from non-dementia. The 6Q-DS demonstrated comparable diagnostic performance to the MMSE and exhibited higher specificity than the AD8.

In our dataset, the feeling of depression emerged as an important feature for distinguishing between VMD and more severe stages of dementia. The relationship between depression and dementia is complex and frequently difficult to disentangle. First, depression may result in substantial cognitive impairment, potentially leading to false-positive dementia diagnoses (ie, pseudodementia).^36^ Second, depression may represent a prodromal or early symptom in the trajectory of dementia progression.^36-38^ Third, depression during mid- or late-life may serve as a risk factor for subsequent development of dementia.^39^ Finally, depression may be a psychological response to the awareness of declining cognitive function. Despite this close association, depressive symptoms are often overlooked by widely used dementia screening tools such as the MMSE. Therefore, there is a need for a screening instrument capable of detecting both cognitive impairments and depressive symptoms in individuals at risk for dementia.

In terms of clinical applicability, the 6Q-DS may be especially useful at earlier stages of the patient journey. It can be applied in community-based screening programs, primary care, and general outpatient clinics where consultation time is limited and trained neuropsychologists are not always available. In these settings, the tool may help identify individuals with possible very mild or mild dementia who warrant further comprehensive neuropsychological assessment and etiological investigation. In addition, because the 6Q-DS requires only a brief face-to-face interview, it may be implemented opportunistically during routine chronic disease follow-up visits, annual health examinations, or memory complaints reported by patients or family members. It is intended to complement, rather than replace, full diagnostic work-ups in specialized memory clinics, and may function as a rapid first-line triage tool within the dementia care pathway.

Although the 6Q-DS demonstrated high sensitivity and specificity in identifying individuals with very mild dementia (CDR 0.5), its application in real-world clinical settings requires further scrutiny. In practice, clinicians are not faced with clearly delineated groups of cognitively normal individuals and those with very mild dementia. Instead, the diagnostic challenge lies in broadly identifying dementia across various stages, including both CDR 0.5 and CDR 1. Moreover, like many tools developed in controlled environments, the current study relied on selectively sampled participants, which may not reflect the heterogeneity and complexity of real-world clinical populations.

This study also has several limitations that warrant consideration. First, it was conducted in a single hospital-based setting with participants exclusively from a Chinese population, which may limit the generalizability of findings to other healthcare systems and cultural contexts. Second, we did not stratify model performance by demographic factors such as age or educational level, both of which can influence cognitive screening accuracy. Third, the majority of participants had AD, leaving the predictive validity of the 6Q-DS for other dementia subtypes uncertain. Fourth, due to copyright restrictions, an estimated version of the MMSE was used, preventing a direct comparison with the original MMSE. Fifth, the 6Q-DS assesses only limited cognitive domains—memory, temporal orientation, and concentration—while omitting key functions such as episodic memory, executive function, and working memory, which are often impaired in early-stage dementia.^40^ While comprehensive neuropsychological assessments are more sensitive to such subtle deficits,^41^ their lengthy administration time limits their feasibility in routine practice.

Taken together, these limitations highlight the need for future large-scale validation of the 6Q-DS across diverse populations, clinical settings, and dementia subtypes. Further studies should explore its diagnostic utility across different age and education levels and examine integration with complementary diagnostic systems—such as facial asymmetry detection and joint movement analysis—to enhance screening performance and practical utility in real-world environments.

### Conclusion

The present study developed and evaluated the 6Q-DS, a brief machine learning–based screening tool for the identification of dementia in older adults. The 6Q-DS demonstrated reasonable performance in distinguishing individuals with dementia from those without, particularly in cases of mild to moderate severity. It also showed high sensitivity in detecting very mild dementia. Given its brevity, simplicity, and ease of administration, the 6Q-DS holds promise for routine use in clinical settings. As a rapid, first-line screening instrument, it may support the early detection of dementia and facilitate timely referral for comprehensive diagnostic evaluation.

## Data Availability

The data that support the findings of this study are available from the corresponding author, upon reasonable request.*
